# Hyperaldosteronism from a large adrenal adenoma in a patient with bilateral adrenal nodules

**DOI:** 10.1002/ccr3.2560

**Published:** 2019-12-04

**Authors:** Sreedhar Adapa, Venu Madhav Konala, Srikanth Naramala, Hemant Dhingra, Sean W. Tower, Prem Sahasranam, Fan Zhou, Olayinka Omololu, Frank Gavini, Russell R. Martin

**Affiliations:** ^1^ Saint Agnes Medical Center Fresno CA USA; ^2^ Ashland‐Bellefonte Cancer Center Ashland KY USA; ^3^ Adventist Medical Center Hanford CA USA; ^4^ Visalia Medical Clinic Inc Visalia CA USA

**Keywords:** adrenal nodule, hypokalemia, primary aldosteronism, resistant hypertension, secondary hypertension

## Abstract

Primary aldosteronism (PA) is a potentially reversible cause of uncontrolled hypertension. Early diagnosis and timely management of PA can prevent end‐organ damage. Aldosteronoma Resolution Score (ARS) is a useful tool to predict cure rates and resolution of hypertension after adrenalectomy.

## Graphical abstract

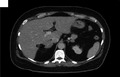

## 1 INTRODUCTION

Primary aldosteronism (PA) is one of the most common causes of secondary and resistant hypertension that can be potentially reversible. The classic presentation of resistant hypertension with hypokalemia is encountered in 10%‐30% of patients with PA. Most of the patients with PA have bilateral adrenal hyperplasia and are normokalemic. We present a case of a 52‐year‐old caucasian female with severe hypokalemia and resistant hypertension. The hypokalemia was very resistant to the treatment and was needing 150‐200 meq of potassium supplements daily. Biochemical workup was significant for an elevated aldosterone‐renin ratio, which was confirmed by high urinary aldosterone level. Imaging revealed bilateral adrenal nodules with right larger than the left. Adrenal vein sampling lateralized to the right, and the patient underwent robotic right adrenalectomy. Postoperatively, patient became normokalemic with a decrease in blood pressure medications and normalization of aldosterone‐renin ratio.

Primary aldosteronism results from bilateral adrenal hyperplasia also known as idiopathic hyperaldosteronism (IHA) in 60% of patients, unilateral aldosterone producing adenoma (APA) in 30% of patients and rest of the causes accounting for 10% of patients.^1^

## 2 CASE REPORT

A 52‐year‐old caucasian female presented to the emergency room with a chief complaint of inability to stand along with gradual onset of fatigue, palpitations, tachycardia, and muscle weakness for 7 days. Patient was taking more potassium supplements because of frequent episodes of muscle weakness. The patient denied any gastrointestinal symptoms. She had previous intermittent episodes of hypokalemia. Her home medications include amlodipine 10 mg daily, olmesartan 40 mg daily, chlorthalidone 25 mg daily, metoprolol extended release (ER) 50 mg daily, levothyroxine 50 mcg daily, paroxetine 50 mg daily, and multivitamin one tablet daily. Two weeks before admission, the patient's antihypertensive regimen was changed from spironolactone to chlorthalidone. The vital signs in the emergency room were blood pressure 160/85 mm Hg, pulse rate 84 beats per minute, respiratory rate 18 breaths per minute, and temperature 36.1°C. Physical examination was unremarkable.

Electrocardiogram revealed sinus tachycardia at 106 beats per minute, left axis deviation, left ventricular hypertrophy with secondary repolarization abnormality. Corrected QT interval was normal with no T wave abnormalities and absence of U wave. Initial laboratory data summarized in Table 1. The hypokalemia was very resistant to the treatment and needed 150‐200 meq of potassium supplements daily. The 24 hours urine potassium level was 550 meq (normal level 23‐126 meq) consistent with renal wasting of potassium, and primary aldosteronism was suspected. The screening test was performed, renin activity 0.1 ng/mL/hr, aldosterone level 22.8 ng/dL, and aldosterone‐renin ratio (ARR) was 228 (normal <25). All samples were obtained in the morning and in seated position. The 24 hours urine aldosterone level was checked to confirm the hyperaldosteronism, which resulted in level 87 mcg/day (normal 1.2‐28.1).

**Table 1:** Pertinent laboratory data

Laboratory test	Patients value	Normal reference range
Hemoglobin	13.6 gm/dL	12‐16 gm/dL
White cell count	13.4 × 10^3^ cells/cubic mm	4‐11 cells/cubic mm
Platelets	499 × 10^3^ cells/cubic mm	499 × 10^3^ cells/cubic mm
Sodium	138 mmol/L	135‐144 mmol/L
Potassium	1.8 mmol/L	3.5‐5.1 mmol/L
Chloride	87 mmol/L	101‐111 mmol/L
Bicarbonate	36 mmol/L	22‐32 mmol/L
Glucose	140 mg/dL	74‐118 mg/dL
Blood urea nitrogen	13 mg/dL	6‐20 mg/dL
Creatinine	0.6 mg/dL	0.5‐1.2 mg/dL
Magnesium	1.8 mg/dL	1.8‐2.5 mg/dL
Calcium	9.6 mg/dL	7.5‐10.4 mg/dL
Albumin	4.4 g/dL	3.5‐5.7 g/dL
Thyroid‐stimulating hormone	4.74 milli‐international units per L	0.4‐5.6 milli‐international units per L
Renin activity supine	0.1 ng/mL/hr	Supine 0.2‐1.6 ng/mL/hr
Aldosterone supine	22.8 ng/dL	Supine <= 16 ng/dL
Aldosterone‐renin ratio	225	<25
24 h urine aldosterone level	87 mcg/day	1.2‐28.1 mcg/day
Free metanephrine	<25 pg/mL	<=57 pg/mL
Free normetanephrine	85 pg/mL	<=148 pg/mL
Plasma adrenocorticotropic hormone	11 pg/mL	6‐50 pg/mL
Dehydroepiandrosterone	83 mcg/dL	8‐188 mcg/dL

**Figure 1:**
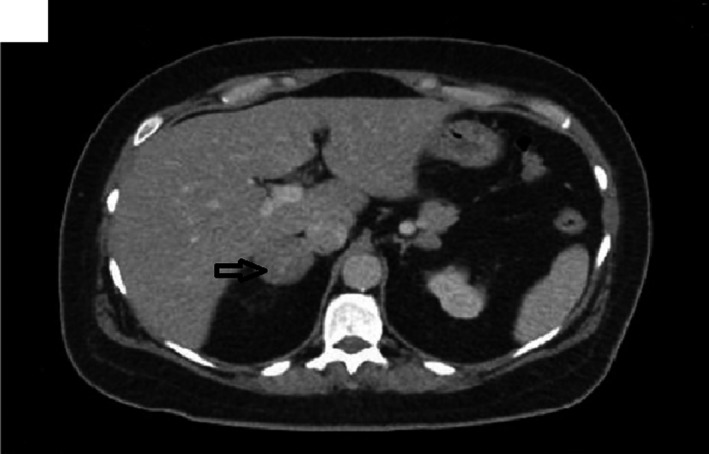
CT of the abdomen with contrast showing 4 cm right adrenal adenoma

**Figure 2:**
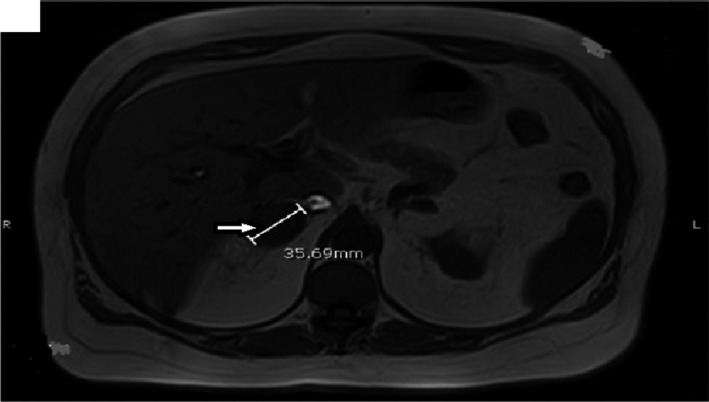
Magnetic resonance imaging (MRI) abdomen with and without contrast revealed a 3.6 cm right adrenal mass

Computerized tomography (CT) of abdomen and pelvis with contrast showed bilateral adrenal gland nodules measuring up to 4 cm in size, nonspecific for adrenal adenomas versus adrenal metastases (Figure 1). For characterization of the adrenal adenoma, high resolution CT of abdomen or magnetic resonance imaging (MRI) of abdomen with and without contrast was recommended. Magnetic resonance imaging abdomen with and without contrast revealed a 3.6 cm right adrenal mass, which was well‐circumscribed and demonstrated a significant signal dropout on out of phase imaging essentially diagnosing an adrenal adenoma (Figure 2). A small left adrenal 1 cm nodule also had the appearance consistent with an adrenal adenoma.

The patient was discharged from the hospital with a potassium level of 3.6 mmol/L. The discharge medications were triamterene 50 mg daily, potassium chloride 40 meq three times a day, amlodipine 10 mg daily, metoprolol ER 50 mg daily, and olmesartan 20 mg daily. The patient was referred for adrenal vein sampling (AVS) to lateralize the aldosterone secretion.

**Figure 3:**
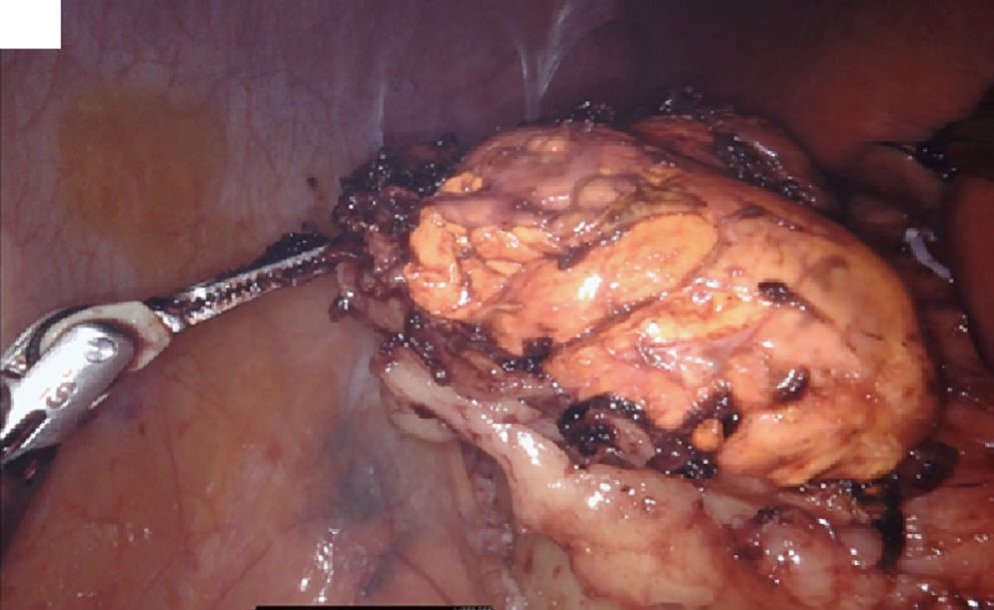
Surgically removed the right adrenal gland

**Figure 4:**
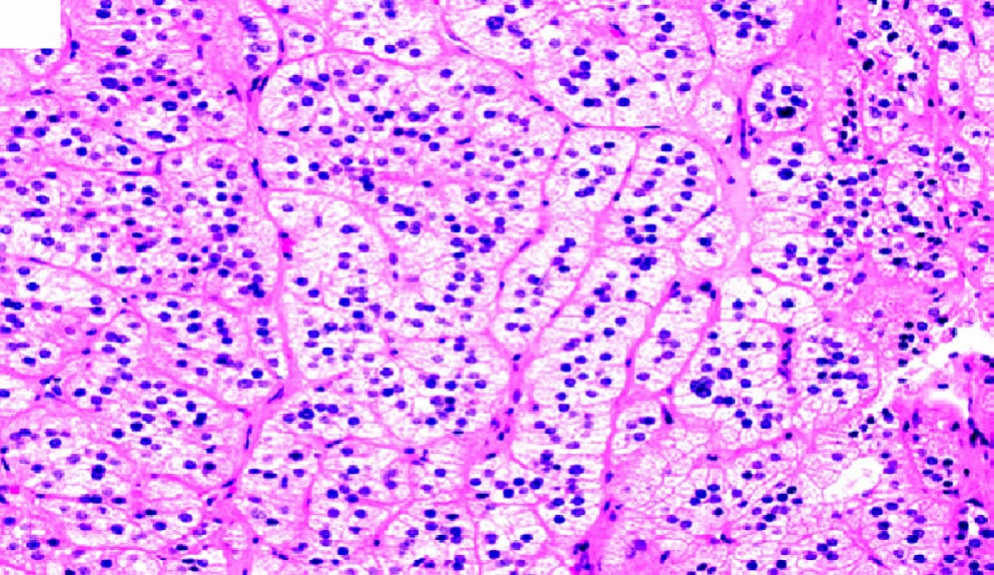
Classic benign adrenal cortical adenoma histomorphology showing characteristic microvascular cytoplasm mimicking the adrenal zona fasciculata. (hematoxylin‐eosin, original amplification ×200)

**Figure 5:**
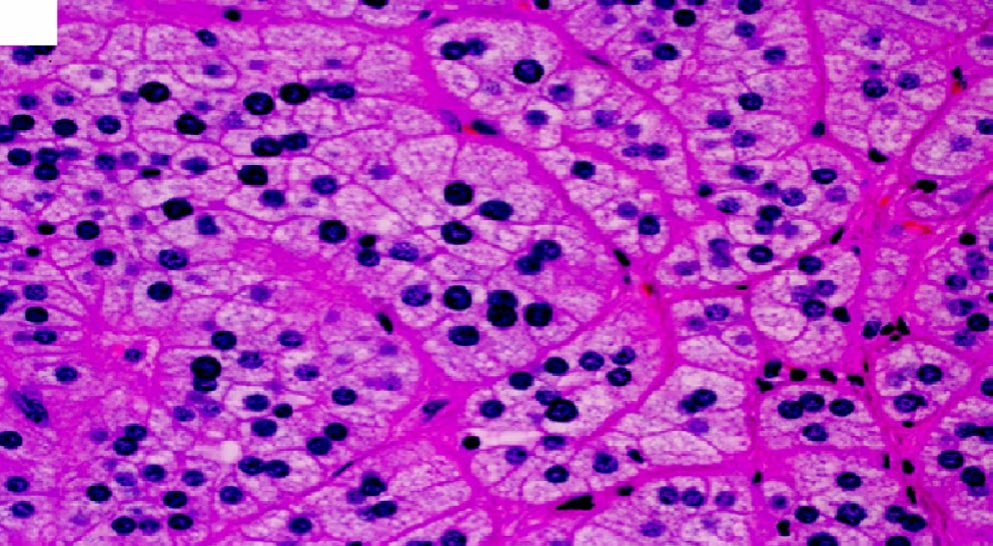
Adrenal cortical adenoma showing characteristic microvascular cytoplasm mimicking the adrenal zona fasciculata. (hematoxylin‐eosin, original amplification ×400)

**Figure 6:**
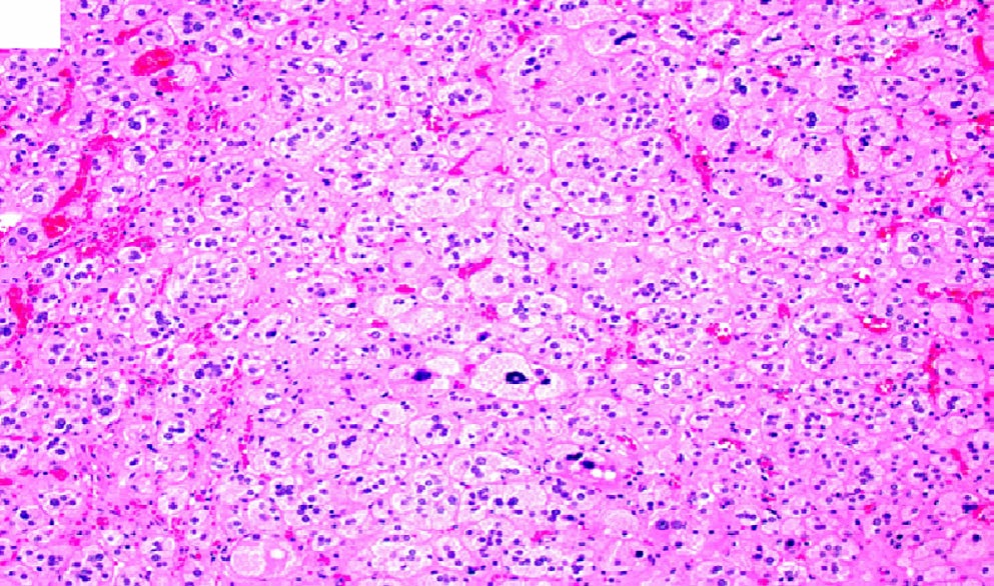
Benign adrenal cortical adenoma histomorphology showing degenerative nuclear atypia, occasional multinucleation. (hematoxylin‐eosin, original amplification ×200)

**Figure 7:**
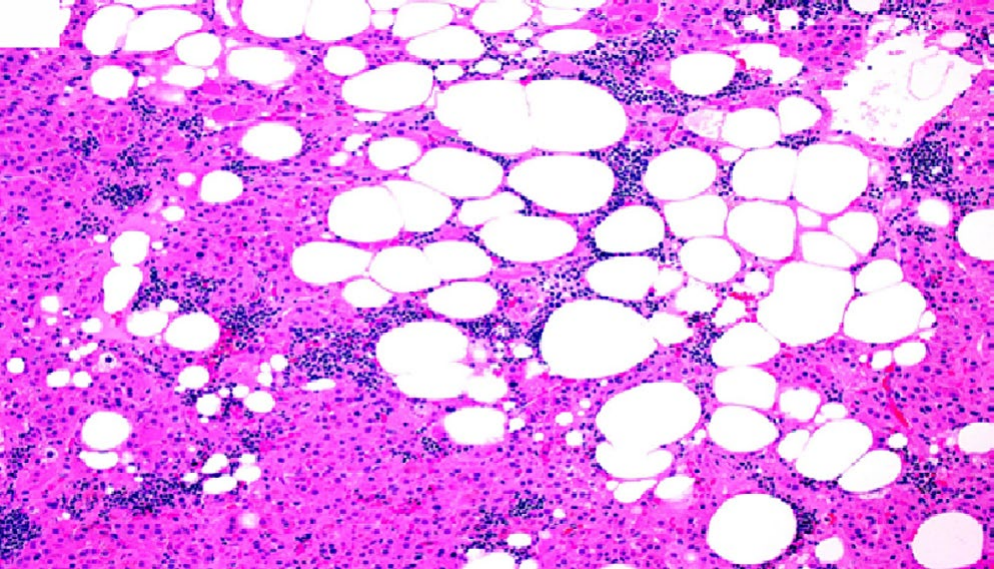
Benign adrenal cortical adenoma histomorphology showing lipid‐poor/eosinophilic tumor cells with fatty infiltrate and lymphocytic infiltrate within the adenoma. (hematoxylin‐eosin, original amplification ×200)

**Figure 8:**
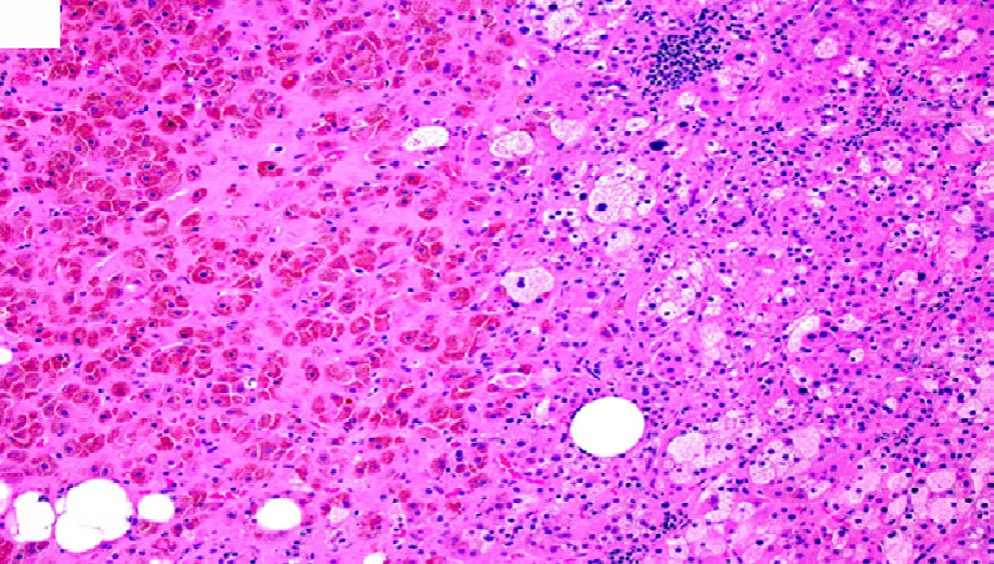
Benign adrenal cortical adenoma histomorphology showing mixed lipid‐poor and lipid‐rich tumor cells with lymphocytic infiltrate on the right side of the image. Please note the pigmented adrenocortical adenoma cells on the left side of the image. (hematoxylin‐eosin, original amplification ×200)

**Figure 9:**
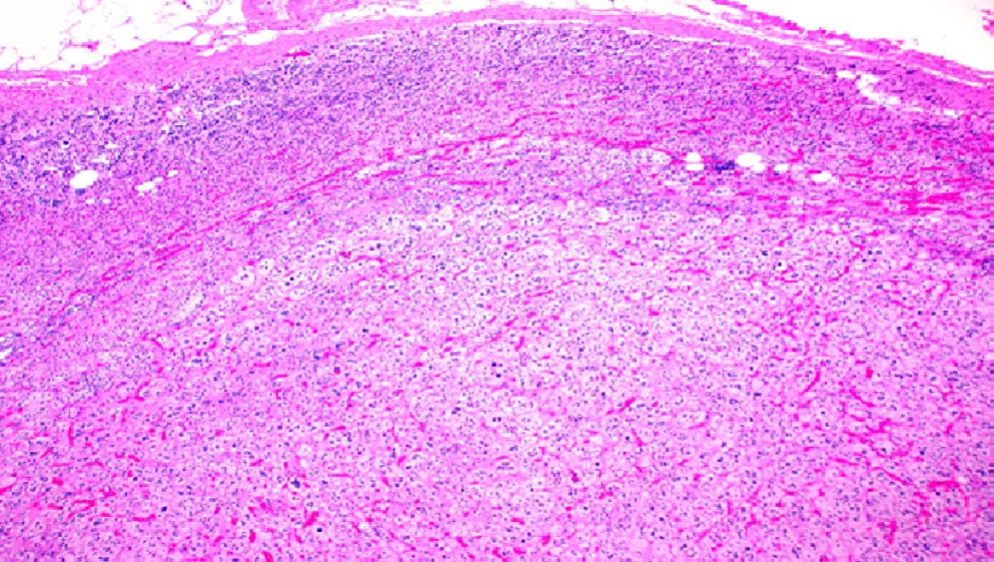
Benign adrenal cortical adenoma histomorphology showing the tumor pushing the adjacent normal cortex away without forming distinct demarcation or fibrous capsule. (hematoxylin‐eosin, original amplification ×100)

**Figure 10:**
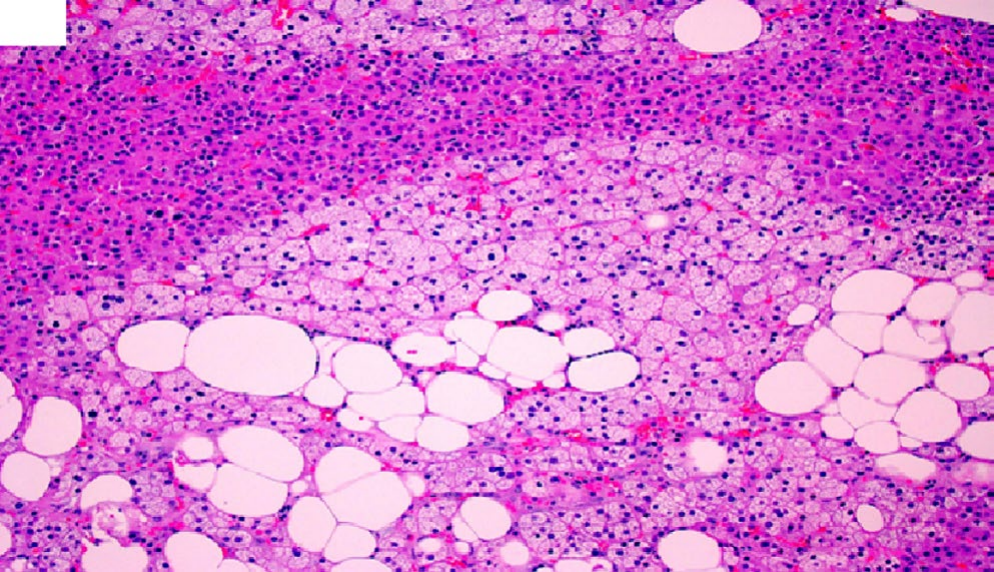
Benign adrenal cortical adenoma histomorphology showing lipid‐poor/eosinophilic tumor cells with fatty infiltrate and lymphocytic infiltrate within the adenoma. (hematoxylin‐eosin, original amplification ×200)

Adrenal vein sampling revealed a striking elevation of aldosterone from the right adrenal vein, 37 times that of peripheral blood and mild elevation of aldosterone from the left adrenal vein, 3 times that of peripheral blood. The patient was referred to the endocrine surgeon for robotic adrenalectomy. The patient underwent robotic right adrenalectomy, and right adrenal gland measured approximately 6 × 4 × 4 cm (Figure 3). Pathologic description of surgically resected mass shown from Figures 4, 5, 6, 7, 8, 9, 10.

One month after the right adrenalectomy, patient's potassium was 3.9 mmol/L and was not taking any potassium supplements. ARR was in the normal range 14.4 (0.9‐28.9). The 24 hours urine aldosterone level after surgery was 16.4 mcg/day. The patient needed only amlodipine and metoprolol to control the blood pressure. The patient is being followed closely in the clinic with a plan to get biannual MRI to evaluate for further left adrenal gland enlargement.

## 3 DISCUSSION

Conn first described PA in a patient manifesting with resistant hypertension and hypokalemia in 1955.^2^ PA affects 5%‐18% of adults with hypertension and 14%‐23% with resistant hypertension.^3^, ^4^ The classic presentation of resistant hypertension with hypokalemia is encountered in 10%‐30% of patients with PA. Most of the patients with PA are normokalemic as per recent evidence.^3^, ^5^

Pathophysiologically aldosterone regulates blood pressure by increased sodium absorption through thiazide sensitive sodium‐chloride channels in distal convoluted tubule (DCT), amiloride‐sensitive epithelial sodium channel (ENac) in collecting duct, thus increasing sodium load. It also increases vascular reactivity to the vasoconstrictors, endothelin and decreases vascular sensitivity to vasodilators. Aldosterone also stimulates the sympathetic nervous system and increases central blood pressure.^4^ Excess aldosterone production results in potassium wasting, which is compensated by the potassium retaining effect of hypokalemia and thus balancing the potassium concentration at a lower level. Addition of diuretic therapy will induce severe hypokalemia as in our patient.

Evidence has suggested that end‐organ damage is more pronounced with PA compared with other causes of secondary hypertension or essential hypertension. The role of aldosterone in end‐organ damage is independent of hypertension and is driven through perivascular inflammation, vascular remodeling, and fibrosis. The adverse effects resulting from aldosterone excess were significantly reduced by medical or surgical management compared to patients with essential hypertension.^1^ In a retrospective study done in patients with PA compared to patients with essential HTN, it demonstrated increased incidence of stroke, nonfatal MI, and atrial fibrillation in patients with PA with no difference noted in the PA subtypes.^6^ Another study showed decreased proteinuria in patients with PA treated with spironolactone or adrenalectomy compared to patients with essential hypertension.^7^

The screening for PA should be considered in patients with resistant hypertension, hypertension with hypokalemia, hypertension with adrenal incidentaloma. Resistant hypertension is defined as uncontrolled blood pressure despite the concurrent use of three different classes of antihypertensives, including a diuretic, calcium channel blocker, and renin‐angiotensin system inhibitor.^8^ Screening test for PA is plasma aldosterone‐renin ratio (ARR) that identifies excess plasma aldosterone production and suppression of plasma renin activity. Many drugs like beta‐blockers, renin‐angiotensin system inhibitors, selective serotonin reuptake inhibitors, and oral contraceptives interfere with the assay and should be ideally discontinued 2 weeks before testing.^1^ Studies have shown that spironolactone will interfere with the ARR and should be discontinued 4 weeks prior to testing.^9^ Other factors that influence the tests are age, smoking, renal function, posture resulting in false negative, and positive results. Many studies recommended ARR of 20‐40, while the ratio of 35 has 100% sensitivity and 92% specificity in diagnosing PA.^1^

The recently updated endocrine guidelines continue to recommend the use of aldosterone‐renin ratio for screening. A value higher than 30 merits investigation. A confirmatory test is not required in clear cut cases like in our patient when there is spontaneous hypokalemia undetectable renin levels and plasma aldosterone concentration greater than 20 ng/dL.^10^ The patient also had elevated 24 urine aldosterone levels and hence required no dynamic confirmatory test. Oral salt loading test, saline infusion test, fludrocortisone suppression test, captopril suppression test, and 24‐hours urine for aldosterone levels are the confirmatory tests. Patients with uncontrolled hypertension, underlying cardiac, and renal impairment should avoid salt loading and saline infusion tests. No single test is superior compared with the other. We must consider availability and cost as well as patient compliance, comorbidities, and medications while choosing the specific confirmatory test.^1^, ^4^

Imaging and adrenal vein sampling (AVS) establishes the etiology and localization of the lesion for PA. The endocrine society clinical practice guidelines recommend in patients who are diagnosed with PA and considered for surgical resection, bilateral AVS should be performed in all patients.^10^ Adrenal CT scan is the initial study in identifying the subtypes of PA and guides interventional radiologist and surgeon for further management.^10^

In a study by Kempers et al CT/MRI showed lateralization in 37.8% patients that had conflicting results on AVS.^11^ Further, using imaging solely for localization, 19% could have been excluded inappropriately from adrenalectomy, and 14.6% of patients could have had surgery inappropriately. In patients with PA, AVS is the gold standard test to differentiate unilateral (APA, UAH) from bilateral (IAH) disease.^10^, ^11^

In patients with aldosterone hypersecretion bilaterally or poor surgical candidates, medical management with mineralocorticoid (MR) receptor antagonist is recommended.^10^, ^12^ Spironolactone and eplerenone are the MR antagonists used in clinical practice. Eplerenone is a more specific inhibitor of aldosterone and less toxic compared with spironolactone, which is cheaper, widely available, and more potent.^13^ Blood pressure takes several months to normalize but hypokalemia resolves immediately.

Surgical management will involve adrenalectomy for the secretion of aldosterone due to unilateral APA. Lateral transperitoneal laparoscopic approach and retroperitoneal laparoscopic approach are used for adrenalectomy with no significant difference in outcomes on several meta‐analysis.^14^, ^15^ Robotic‐assisted adrenalectomy is being preferred to laparoscopic approaches because it is equally safe with minimal blood loss and shorter hospital stay.^16^

The medical and surgical management of PA is very effective resulting normalization of hypokalemia and aldosterone levels in all the patients. Studies have shown that adrenalectomy reduced arterial stiffness, left ventricular hypertrophy, and also reversed myocardial fibrosis.^1^

Aldosteronoma Resolution Score (ARS) by Zarnegar et al predicts post adrenalectomy resolution of hypertension in patients with APA with preoperative clinical variables as mentioned in Table 2.^17^

**Table 2:** Aldosteronoma resolution score (ARS)^17^

Variables	Points
Female sex	1
Duration of preoperative hypertension less than 6 y	1
Number of preoperative antihypertensive medications ≤2	2
BMI ≤ 25 kg/m^2^	1
ARS of 0‐1—low chance of hypertension resolution post adrenalectomy	
ARS 4‐5—high chance of hypertension resolution post adrenalectomy	

Aldosteronoma Resolution Score predicted the cure rates and complete resolution of hypertension with about a quarter of the patients in the low‐score group (0‐1) and three‐fourths in the high‐score group (4‐5) in the above study. Aldosteronoma Resolution Score model was confirmed as a presurgical clinical tool in Japanese population by Utsumi et al.^18^

## 4 CONCLUSION

Our case demonstrates the classic presentation of primary aldosteronism with hypokalemia and resistant hypertension. Adrenal vein sampling played a very critical role in our patient in lateralizing the hypersecretion of aldosterone when presented with bilateral adrenal masses and in guiding appropriate management. Although the right adrenal gland was large, the tumor was benign, and the largest reported in the literature was 7 cm.^19^ Appropriate management resulted in the resolution of primary aldosteronism in our patient.

## CONFLICT OF INTEREST

The authors declare no conflict of interests.

## AUTHOR CONTRIBUTIONS

SA, SWT, PS, FN, OO, and RRM: directly involved in management of the patient. SA, VMK, SN, HD, and FG: involved in preparation of manuscript and discussion. FN: provided pathology images. RRM: provided surgical image.

## ETHICAL APPROVAL

This is a case report, so exemption has been provided.

## INFORMED CONSENT

Both informed consent and written consent were obtained from the patient.
